# Homœopathic Treatment of Toothache from Pulp-Capping

**Published:** 1894-08

**Authors:** Charles H. Taft

**Affiliations:** Chicago, Ill.


### HOMŒOPATHIC TREATMENT OF TOOTHACHE FROM PULP-CAPPING.

BY CHARLES H. TAFT, D.M.D., CHICAGO, ILL.

The interest which has been awakened in the homœopathic treatment of cases occurring in the every-day practice of the dentist, as manifested by the numerous letters I have received requesting such information as I could give and which would enable others to pursue a line of study intelligently, with the end in view of having at command, if nothing more, an effectual method of quickly relieving the various kinds of toothache, from causes with which we are all more or less familiar, prompts me to relate a recent case in practice, and to suggest some of the indications which furnish a key-note for the remedy given.

As a preface to the subject I have chosen, I may say that in recent papers I have dwelt at some length upon the philosophy of the homœopathic law of cure, and have suggested the work entitled "Hering's Domestic Physician," with its chapter upon the affections of the teeth, as furnishing, to me at least, an invaluable aid for the treatment of many cases where the ordinary dental therapeutic agents at our command have proved either wholly unsatisfactory or unsuccessful.

It gives me pleasure to know that many others in our profession are working along the lines I have suggested, although none can appreciate more fully than myself how greatly one is handicapped in such study and its practical application by those who have had the advantages of a thorough medical training.

A complete mastery of the art of healing or the ability to relieve human suffering, even when based upon a simple and well-established law, is not to be acquired in a day, if indeed in a lifetime; but to the earnest and thoughtful student, neophyte though he be, there is a fascination in the study of this law and the manifestations of its operation which cannot fail to urge one on to still greater effort, when once such study has been entered upon free from all partisan prejudice and with the simple desire to find the truth.

I am well aware that many practitioners make it a practice never to cap an exposed pulp, even though the pulp is a freshly exposed one and devoid of any inflammation or irritation from whatever cause, but deem it best to destroy it at once. My own experience in any case is to destroy it only when I am confident its vitality cannot be successfully restored and maintained without giving the patient future and continual annoyance; for I am of the opinion that a live tooth, like any other organ, is an infinitely better member of the economy than is one in which such vitality has been destroyed, and especially if the tooth happens to be one of the incisors or bicuspids. But this is simply an individual opinion, based upon experience and a careful treatment of such cases.

The following case in practice is intended to furnish one of the many reasons for such belief, and to suggest such therapeutic treatment as a similar emergency may demand.

On March 16, Mr. F., aged thirty-two, came to my office for a series of appointments. The rubber dam was adjusted, a large corono-distal cavity in the right superior second bicuspid and another in the corono-mesial surface of the right superior first molar were carefully excavated, but not without avoiding an exposure of the pulp in the molar, and perilously near one in the bicuspid. Both teeth were exquisitely sensitive to the touch of an excavator, but had never given the patient the slightest discomfort. The exposed pulp was capped according to the method I invariably employ, by applying oil of clove to the pulp and dusting over it a sufficient amount of dry oxide of zinc. The same precaution was taken with the bicuspid, and both cavities were then filled with oxyphosphate cement, mixed to the proper consistency. The operation finished, the rubber dam was removed; the patient said he felt no pain nor discomfort from the fillings, and, rinsing out the mouth, was about to leave the chair, when he seized his head with both hands and cried aloud with pain.

The pain continued for several minutes, coming in terrific paroxysms of but a few seconds apart, with determination of blood to the head and face. Becoming well-nigh frantic with pain, he implored me to extract the tooth at once and in no uncertain tone, saying that he simply could not and would not stand it.

There were but two or three expedients,—the first, to comply with his imperative command; the second, to remove the filling and apply arsenous acid; and the third, to give the one, and only one, plainly indicated homœopathic remedy which the symptoms called for.

Watching his symptoms for a few moments, I decided upon the last expedient, and placed upon his tongue a few pellets of the potentized chamomilla and waited for its effect.

Between the paroxysms he asked what I had given him, and, contrary to my usual custom of naming the drug, I said it was chamomilla.

With a good knowledge of drugs on his part, being himself in the wholesale drug business, he remarked, with no apparent confidence in me or the remedy, that if chamomilla stopped such suffering as his it would be the first time he ever heard of its so doing. He added, however, before he left the office, that he was open to conviction.

As I stood by the chair, watching him in his writhing, I felt sure I had made no mistake in the selection of the remedy, and that, like an arrow from the bow, it would go straight to the mark. Within five minutes his writhing ceased, and the pain melted away like dew before the sun; his face began quickly to assume its normal color, and the paroxysms only recurred at long intervals and with decreasing severity during the next fifteen minutes, until they ceased entirely. He remained an hour after the medicine had taken effect, fully expecting the pain to return and to compel me to extract the tooth, but the latter was obstinate and positively refused to ache. Finally he left the office, with the assurance from me that in all probability there would be no recurrence of pain, and with an equally strong assurance and belief on his part that he would have to hunt up a dentist in the middle of the night and have him extract the tooth.

What, then, was accomplished in this case? for I am prompted to speak of it for the reason that never but once before in a practice covering a period of eight years do I remember seeing a case where the limit to physical endurance of pain was put to so severe a test. First, the pain was quickly brought under control and then banished entirely without the use of morphine, narcotics, palliatives, or counter-irritants, and without resorting to extraction.

Secondly, the vitality of the tooth is maintained, and, without some future manifestations of discomfort of, let us say, *improbable* occurrence (for the reason that the tooth had never ached, the exposure being a perfectly healthy one, and the capping one of absolute non-irritating substance, under normal conditions), is likely, I believe, from observation and experience with similar cases, to be maintained indefinitely.

Thirdly, the satisfaction which comes from a knowledge of what particular drugs to employ in any given case, and the ability to see, after a careful study of the law of similars and its operation, just *why* chamomilla, for instance, would relieve one kind of toothache and be entirely inoperative or ineffectual in another kind. In other words, recognition of the fact that there can never be any one drug which would act as a specific for all kinds of toothache.

Up to the present writing, during which time the patient has had several subsequent sittings, there has been not the slightest recurrence of pain or discomfort.

Doubtless some sceptical friend will venture the remark that the cessation of pain was not due to the medicine, but ceased of its own accord. Another will probably suggest that I hypnotized him or the tooth. A third will emphatically assert it was not the medicine, for how could attenuated "moonshine" effect so complete a relief? While a fourth will incline to the belief that I employed the methods of the Christian Scientist, and assured him that his suffering was simply a delusion and did not really exist.

To all such my statement will undoubtedly be of little interest or help in similar cases, but to those who are interested in the action of drugs and the laws which govern it, so far as it concerns us in our every-day practice, the paper is especially addressed, with a view to pointing out the fact that one of the strongest indications of chamomilla for the relief of a toothache like the one described is the fact of its driving the patients so nearly *frantic* with pain that it is difficult for them to speak a pleasant word, or in a pleasant tone of voice. In a word, are thoroughly *ugly*.

In striking contrast to the always jovial, happy-go-lucky disposition of the patient was his manner and language towards me during those first fifteen minutes after the operation. That and the sudden rush of blood to the cheeks were two of the strongest indications pointing to chamomilla in preference to other drugs having equally strong indications in other cases, but which, being not homœopathic to the case in question, would have proved equally unavailing and unsuccessful.

Upon the ability to differentiate clearly and carefully between the symptoms in any given case like the above, which plainly point to one drug, and *only* one, depends the success of him who attempts to apply the art of dental medicine in strict accordance with the law of similars.

It was Samuel Hahnemann who said,—

"When we have to do with an art whose end is the saving of human life, any neglect to make ourselves thorough masters of it becomes a crime."

When we, as dentists, have to do with an art one of whose ends is the alleviation of human suffering, should a neglect to make ourselves thorough masters of it become any the less a crime? The question is one that may well command our thoughtful consideration, and must be answered by every man according to his own best judgment and with such methods as he alone has at his command.

